# Effect of MWCNT surface and chemical modification on in vitro cellular response

**DOI:** 10.1007/s11051-012-1181-1

**Published:** 2012-09-12

**Authors:** Aneta Fraczek-Szczypta, Elzbieta Menaszek, Tahmina Bahar Syeda, Anil Misra, Mohammad Alavijeh, Jimi Adu, Stanislaw Blazewicz

**Affiliations:** 1 Department of Biomaterials, Faculty of Materials Science and Ceramics, AGH-University of Science and Technology, Al Mickiewicza 30, 30-059 Kraków, Poland; 2 Department of Cytobiology, Collegium Medicum, Jagiellonian University, Medyczna 9, 30-068 Kraków, Poland; 3 Pharmidex Pharmaceutical Services, 72 New Bond Street, Mayfair, London, W1S 1RR UK; 4 School of Pharmacy & Biomolecular Sciences, University of Brighton, Huxley Building, Brighton, BN2 4GJ UK

**Keywords:** Carbon nanotubes, Surface characterisation, Functionalisation, In vitro investigation

## Abstract

The aim of this study was to evaluate the impact of multi-walled carbon nanotubes (MWCNTs with diameter in the range of 10–30 nm) before and after chemical surface functionalisation on macrophages response. The study has shown that the detailed analysis of the physicochemical properties of this particular form of carbon nanomaterial is a crucial issue to interpret properly its impact on the cellular response. Effects of carbon nanotubes (CNTs) characteristics, including purity, dispersity, chemistry and dimension upon the nature of the cell environment–material interaction were investigated. Various techniques involving electron microscopy (SEM, TEM), infrared spectroscopy (FTIR), inductively coupled plasma optical emission spectrometry, X-ray photoelectron spectroscopy have been employed to evaluate the physicochemical properties of the materials. The results demonstrate that the way of CNT preparation prior to biological tests has a fundamental impact on their behavior, cell viability and the nature of cell–nanotube interaction. Chemical functionalisation of CNTs in an acidic ambient (MWCNT-Fs) facilitates interaction with cells by two possible mechanisms, namely, endocytosis/phagocytosis and by energy-independent passive process. The results indicate that MWCNT-F in macrophages may decrease the cell proliferation process by interfering with the mitotic apparatus without negative consequences on cell viability. On the contrary, the as-prepared MWCNTs, without any surface treatment produce the least reduction in cell proliferation with reference to control, and the viability of cells exposed to this sample was substantially reduced with respect to control. A possible explanation of such a phenomenon is the presence of MWCNT’s agglomerates surrounded by numerous cells releasing toxic substances.

## Introduction

Nanomaterials as the foundation of nanotechnology are increasing in importance due to the needs of different branches of industry and medicine for modern and improved performance products and medical devices. Increasingly frequently, however, one asks oneself whether in addition to the benefits of nanotechnology, it may also be harmful for health. This question relates primarily to nanoparticles such as carbon nanotubes (CNTs), production of which since 1991 has increased rapidly. The interest in CNTs is mainly associated with their unique properties, which make them attractive options in various consumer, medical, and industrial applications (Schrand et al. 2007; Xu et al. 2008; Harrison and Atala 2007; Kim et al. 2011). Opinions on the biocompatibility of CNTs in vitro and in vivo environments are not, however, unequivocal. Some researchers have compared them to the negative effects of asbestos fibres, ordering special care during handling or disqualifying them completely from further use (Jaurand et al. 2009; Poland et al. 2008). Particular caution is advised during contact of CNTs with skin and the respiratory tract (Tong et al. 2009). Other researchers in turn have indicated that nanotubes are biocompatible and have a positive impact on cell growth and proliferation and, therefore, may be used in tissue engineering (Shi et al. 2007; Matsumoto et al. 2007; Fraczek et al. 2008). Additionally, due to their mobility potential in living systems, they may be successfully used as novel drug delivery systems for therapy and diagnosis (Xu et al. 2008; Menard-Moyon et al. 2010). In contrast to observations that nanotubes tend to accumulate in tissues and organs, other experiments have shown that more than 95 % of CNTs are released in the urine from the body within a few hours (Liu et al. 2009; Lacerda et al. 2008; Guoa et al. 2007) or are degraded (Liu et al. 2010). Analysis of the available literature shows both the beneficial and the adverse impact of nanotubes on a living organism and suggests that both parties are right. Moreover, taking into account the diversity of CNTs resulting primarily from methods of their manufacture, the catalysts used, the synthesis conditions and a variety of methods used for evaluation of their toxicity, it is difficult to agree with either view. Many studies indicate that biocompatibility of CNTs in both in vivo and in vitro studies may be attributed to various factors, including their lengths, functionality, their concentration, duration in the living body, catalyst impurity, agglomeration and even the dispersants used to dissolve the nanotubes (Kagan et al. 2005, 2006; Bianco et al. 2005; Sato et al. 2005; Wick et al. 2007; Aillon et al. 2009; Raja et al. 2007; Coccini et al. 2010; Fiorito et al. 2009). It is also important to develop and validate methods to evaluate the toxicity of nanoparticles to compare properly the experimental results between research institutions. Most aspects of CNT toxicity still remain inadequately identified and further long-term research is required.

The objective of this study is to evaluate the impact of MWCNTs differing in surface chemistry, purity and dispersion-degree on cellular response. The effect of differently prepared MWCNTs on the viability of macrophages (RAW 264.7) was analysed.

## Materials and methods

Three types of as-prepared and functionalised MWCNTs were used in this study. The as-prepared MWCNTs examined in this study were provided by NanoAmorUSA. The pristine MWCNTs had diameters in the range of 10–30 nm and were 1–2 μm long.

**Fig. 1:**
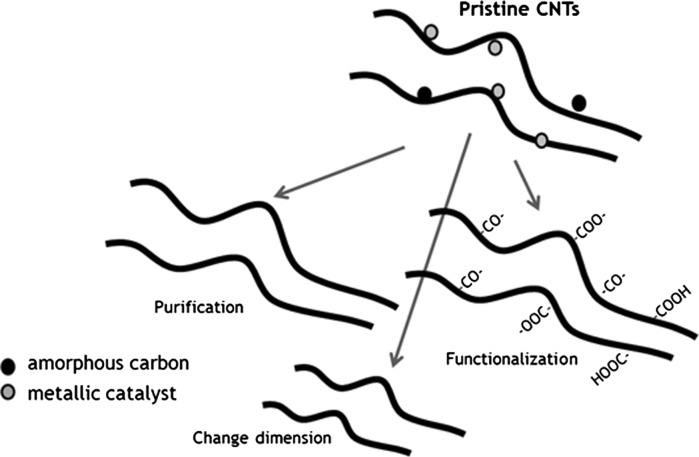
Scheme showing the main goals oxidation of CNTs in a mixture of concentrated acids

The nanotubes were chemically oxidised in a mixture of concentrated H_2_SO_4_ and HNO_3_ acids, according to the procedures described in detail elsewhere (Fraczek-Szczypta et al. 2011). The nanotubes prepared in such a way were referred to as MWCNT-F. The aim of this process was the removal of metallic catalysts and chemical modification of nanotubes by introducing carboxyl acid groups on their surface. Simultaneously, during the oxidation process significant differences in their morphology and length were observed. The possible physical and chemical reactions taking place during the oxidative treatment of CNTs are schematically shown in Fig. 1.

The next step after the chemical oxidation of the MWCNT-F was their functionalisation in ethylenediamine (C_2_H_4_(NH_2_)_2_). Amino-functionalisation of CNTs was performed to verify their influence on cell response. The literature states that the functionalisation of CNTs significantly reduces their cytotoxicity and improves cell growth (Vukovic et al. 2010; Hu et al. 2004).

**Fig. 2:**
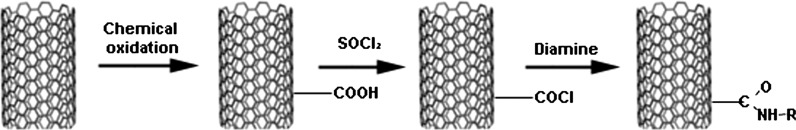
Scheme showing the process of functionalisation of CNTs

This process was performed according to the procedure described elsewhere (Chen et al. 1998; Shen et al. 2007) (Fig. 2).

In the first step, the MWCNT-F were treated in a mixture of SOCl_2_ (thionyl chloride): dimethylformamide (DMF) (20:1) for 24 h at 70 °C, followed by cooling them to room temperature and finally the nanotubes were washed several times using tetrahydrofuran (THF) to remove excess SOCl_2_. The aim of this step was to generate acyl chloride groups and facilitate functionalisation of CNTs with amines. Subsequently, such prepared CNTs were treated with ethylenediamine (C_2_H_4_(NH_2_)_2_) for 48 h at a 95 °C. After this treatment, the CNTs were washed in ethanol and dried under vacuum. CNTs prepared in such a way were denoted as MWCNT-NHs. The morphology of MWCNTs before and after chemical oxidation was analysed using transmission electron microscopy (TEM) (Tecnai G2 F20 (200 kV) and Joel). The absolute zeta potential (ζ) and size distribution of CNTs before and after acid oxidation were performed in phosphate buffer (PBS), using combination of electrophoresis and the LDV technique (*Laser Doppler Velocimetry*, Malvern Zetasizer Nano ZS) in the range of the particle size from 5 nm to 10 μm, with the laser light source of wavelength λ = 520 nm.

The degree of purification of CNTs was determined using inductively coupled plasma optical emission spectrometry (ICP-OES) (Multiwave 3000, Perkin Elmer Co.). Evaluation of the functionalisation of CNTs was performed using Fourier transformation infrared spectroscopy (FTIR) (Bio-Rad FTS60 V spectrometer). The transmission of FTIR spectra was registered in the range of 800–3800 cm^−1^ using KBr pellets.

The study of composition and chemical state of selected elements was made using X-ray photoelectron spectroscopy (XPS) (Vacuum Systems Workshop Ltd., England). Depth of analysis was about 5 nm. Mg Kα X-ray radiation with 200 W energy was used as the excitation source. Electron energy analyzer was set to FAT mode with pass energy 22 eV. The shift of the binding energy due to surface charging effect was calibrated by assuming binding energy of C_1s_ to be always 284.6 eV.

The murine macrophage RAW 264.7 cell line (ATCC, GB) was used in this study. The cells were cultured in 75 cm^2^ tissue culture flasks (Nunc, Denmark) in Dulbecco’s modified Eagle’s medium (DMEM; PAA, Austria) supplemented with antibiotics (penicillin G 100 U/ml, streptomycin 10 μg/ml (Sigma-Aldrich, Germany)) and 10 % bovine foetal serum (PAA, Austria). The flasks of cultured cells were incubated at 37 °C in humidified 95 % air and 5 % CO_2_. Cells were routinely processed by harvesting using a cell scraper and replicated in tissue culture flasks at a ratio of 1:5.

Before incubation with cells (in vitro tests), each type of CNTs was sonicated for 2 min using a tip sonicator (PALMER INSTRUMENTS, Model: CP 130 PB, 130 W power, 20 kHz) in PBS, with a dual concentration of CNT 38 and 80 μg/ml, respectively. Subsequently, CNTs were sterilised by the UV method for 0.5 h. The interaction of nanotubes with RAW 264.7 macrophages was observed using an inverted microscope (Olympus CKX41, Germany) and scanning electron microscopy (SEM, Nova NanoSEM 200, FEI). To determine cytotoxicity of CNTs in contact with macrophages, ToxiLight^®^BioAssay Kit and ViaLight^®^ Plus Kit tests (LONZA Rockland, Inc.) were used. The ViaLight^®^ Plus Kit is intended for rapid and safe detection of proliferation and cytotoxicity of mammalian cells and cell lines in culture by determination of their adenosine triphosphate (ATP) levels. The ToxiLight^®^ BioAssay is a non-destructive bioluminescent cytotoxicity assay designed to measure toxicity in mammalian cells and cell lines in culture. The kit quantitatively measures the release of adenylate kinase (AK) from damaged cells. The cytotoxicity was measured on the third day after seeding.

The results were expressed as mean ± SD obtained from 8 to 11 samples for each experimental group. Significant effects (*p* < 0.05) were determined using the unpaired Student’s *t* test.

## Results and discussion

A strong oxidative environment of acid mixtures has an impact on CNT purity and chemistry. During synthesis of CNTs using both chemical vapour deposition (CVD) and electrical discharge, transition metal catalysts such as Fe, Co, Ni, Mo, etc. are often used. The exceptional ability of transition metals to catalyse CNT formation is primarily linked to their catalytic activity for the decomposition of carbon compounds, their ability to form carbides and the possibility for carbon to diffuse through and over the metals extremely rapidly (Sinnott et al. 1999; Dupuis 2005).

### ICP-OES analysis

Non-purified MWCNTs contained mainly nickel (1.2 wt%). The ICP-OES method indicates that after purification of MWCNTs in acid solution the catalyst residue was reduced to 0.1 wt% for MWCNT-F (Table 1).

**Table 1:** Metal content in the CNTs

Metal content (wt%)	MWCNT	MWCNT-F
Fe	0.3	0.01
Co	0.006	0.001
Ni	1.2	0.1
Al	0.06	0.01

Metals, such as Fe, Ni and Cu, are know to induce the formation of ROS through a Fenton-like reaction and induce intracellular oxidative stress either in a direct or indirect way via extracellular pathways (Kagan et al. 2006; Pulskamp et al. 2007).

### FT-IR spectroscopy

The oxidative treatment of CNTs with the mixture of H_2_SO_4_ and HNO_3_ is a useful technique for removal of impurities and simultaneously to incorporate chemical groups on their surface. All types of nanotubes were characterised by Fourier transform infrared (FT-IR) spectroscopy to identify chemical groups.

**Fig. 3:**
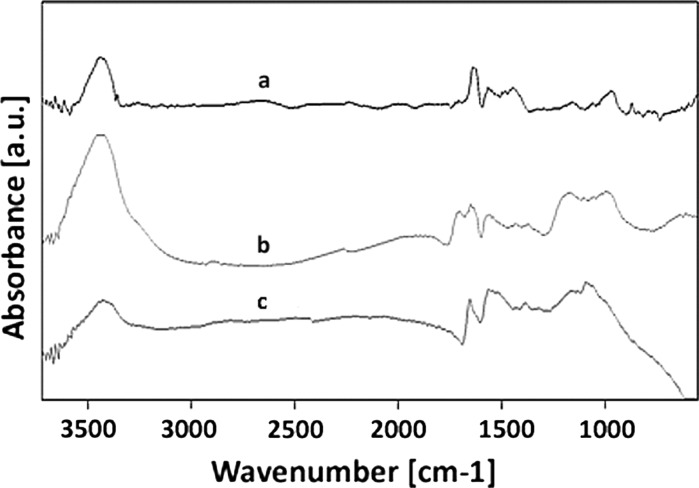
FT-IR spectra of MWCNT (*a*), MWCNT-F (*b*) and MWCNT-NH (*c*)

To clarify the influence of the acid mixture and ethylenediamine on the surface chemistry of MWCNT after the purification and functionalisation processes, the FT-IR investigation was performed and the corresponding results are shown in Fig. 3. The spectrum of the as-prepared MWCNTs shows the C–C stretching bonds in the range of 1,580–1,650 cm^−1^ characteristic to the expected nanotube phonon modes (Fig. 3a). The H_2_SO_4_–HNO_3_ oxidative treatment produces carboxyl groups on the surface of the MWCNTs, as it is indicated by the presence of characteristic bands at 3,439 and 1,710 cm^−1^ (MWCNT-F) assigned to the stretching vibrations of ν(OH) and ν(C=O) of the carboxylic groups (COOH), respectively. The peak at 1379 cm^−1^ was due to sulphate groups ν(OSO_3_H) and δ(OH) bending vibration of COOH. The band at 1,220 cm^−1^ for MWCNT-F was assigned to the ν(C–O) stretching vibration. The increased intensity of the O–H peak after oxidation and the appearance of C=O, C–O and OSO_3_H bonds suggest that oxidation of the CNTs successfully introduced COOH, OH, CO and OSO_3_H groups onto the walls of the nanotubes (Vukovic et al. 2010; Shen et al. 2007). These functional groups are usually found to be attached to the ends of the nanotubes or defects along their wall, due to the enhanced reactivity of these sites (Barros et al. 2005).

The FT-IR absorption spectrum of MWCNT-NH shows a considerably attenuated band at 1,710 cm^−1^. This probably indicates the change of chemical circumstance of C=O in the carboxyl groups due to amino bond formation. It can be observed that the peak of νC=O was moved at 1,653 cm^−1^ for MWCNT-NH. Ethylenediamine functionalisation of MWCNT-F was further evidenced by the characteristic amine peaks. The presence of the band especially at 1,566 cm^−1^ is the most important one to characterise the chemical state of N for amine and should be ascribed to δN–H. Moreover, the C–N stretching vibration peak of ethylenediamine was observed at 1,094 cm^−1^ for MWCNT-NH. The broad band at 3,300–3,600 cm^−1^ was due to the NH_2_ stretch of the amine group. The FT-IR results verify that amines were covalently attached to the MWCNTs (Chen et al. 1998). The presence of the chemical group on CNT’s surface confirms the effectiveness of functionalisation methods.

### XPS

XPS is a useful method to analyse the surface chemistry of CNTs. Using this method the carbon (C_1S_), oxygen (O_1S_) and nitrogen (N_1S_) were detected in all samples, before and after chemical treatments. The element content on the MWCNTs surface was calculated from the peak area of the XPS of each element. The elemental analysis and atomic ratios for all samples are presented in Table 2.

**Table 2:** XPS spectrum of the MWCNTs after purification (MWCNT-F) and functionalisation process (MWCNT-NH)

Carbon nanotubes	Atomic ratio (%)			O/C atomic ratio
	C	O	N	
MWCNT	93.62	6.38	–	0.07
MWCNT-F	87.00	12.37	0.60	0.14
MWCNT-NH	92.67	4.67	2.65	0.06

**Fig. 4:**
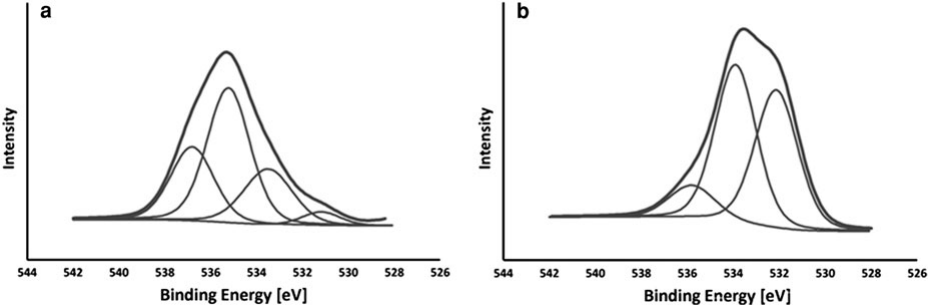
XPS spectra for MWCNT **a** and MWCNT-F **b**

The observed increase of oxygen (O_1S_) and decrease of carbon (C_1S_) for MWCNT-F after chemical purification to 12.37 and 87.00 %, respectively, in comparison with as-prepared MWCNT indicates that functionalisation of the MWCNTs is effective. A similar relationship was also observed by other authors (Kitamura et al. 2011). In the as-prepared MWCNTs, four oxygen (O_1S_) bonds (Fig. 4a) can be distinguished, i.e. at 530.6 eV corresponding to oxidation of nickel into NiO, at 532.9 eV corresponding to functionalised oxygen-containing groups, such as carboxylate, hydroxyl, quinoneand lactone (Ohta et al. 1974; Vigolo et al. 2010), at 534.6 eV attributed to adsorbed oxygen or hydroxyl groups (alcohols, phenol, hydroquinone) (Vigolo et al. 2010) on the CNTs surface and at 536.2 eV.

The MWCNT after chemical purification in the acid mixture (MWCNT-F) contained three oxygen-containing bonds (O_1S_) (Fig. 4b). The peak at 530.6 eV is completely removed from the sample in comparison with as-prepared MWCNT. The absence of this peak indicates that the metal catalyst residue is removed from the sample. After chemical oxidation, the concentration of metal catalysts is significantly reduced in CNTs, which was also confirmed by the ICP method. Moreover, a significant difference between MWCNT and MWCNT-F was observed due to the presence of the peak located at around 532.9 eV. The oxygen atomic content for this peak deduced from XPS analysis of MWCNT-F was almost twice as high (42.30 %) as compared to MWCNT (22.21 %), which confirms the effectiveness of functionalisation methods of their surfaces. Additionally, a higher nitrogen content is observed for nanotubes after functionalisation in ethylenediamine (MWCNT-NH) in comparison with MWCNT and MWCNT-F (Table 1). The increasing content of nitrogen (N_1S_) with the decreasing oxygen concentration (O_1S_) for MWCNT-NH in comparison with MWCNT-F suggested the presence of amine groups on the surface of CNTs. The analysis of a broad C_1S_ peak shows that its component at around 289 eV attributed to sp^2^ carbon bound to two oxygen atoms (O–C=O) both for MWCNT-F and MWCNT-NH are different. Decreasing the relative concentration of carboxylate acid groups for MWCNT-NH (5.20 %) in comparison with MWCNT-F (7.17 %) might suggest that they are converted to amine groups.

### Morphology and structure of CNTs

The differences in morphology of MWCNTs before and after acid oxidation were observed. The observed changes in MWCNT-Fs in comparison to the as-prepared MWCNT are connected with decreases in length.

**Fig. 5:**
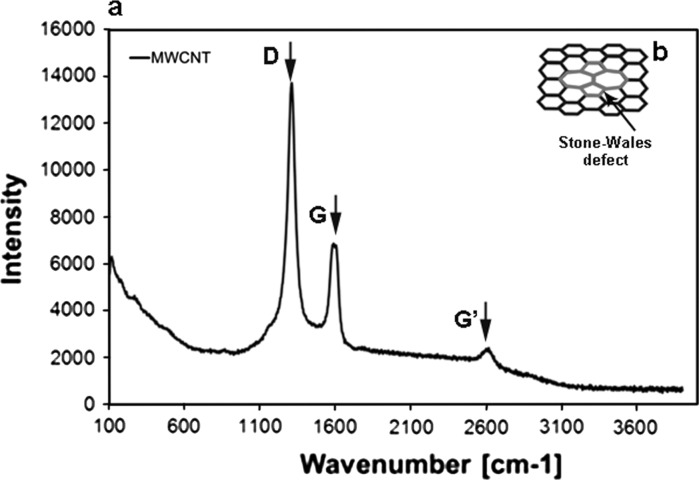
Raman spectra of MWCNTs (**a**), Stone–Wales (SW) defect (**b**)

Shortening the length of the MWCNT-Fs is related to the presence of defects, especially the topological defects such as Stone–Wales (SW) transformation. In general, the term defect means any deviation from the hexagonal arrangement of carbon atoms in a graphene layer. Topological defects (Stone–Wales) are the most probable type of defects from the standpoint of the properties of nanotubes and are related to the presence of two pentagonal and heptagonal rings (Fig. 5b) (Stone and Wales 1986; Dinadayalane and Leszczynski 2007). Due to the presence of such defects in the graphene surface layer of nanotubes, such sites are thermodynamically unstable, and thus, particularly prone to strong oxidising agents. Moreover, the pristine CNTs possess other defects such as a disordered structure or lattice defects in the graphite structure which affect their stability. The presence of such defects in the graphite structure of MWCNTs was found by Raman spectroscopy (Fig. 5a).

**Fig. 6:**
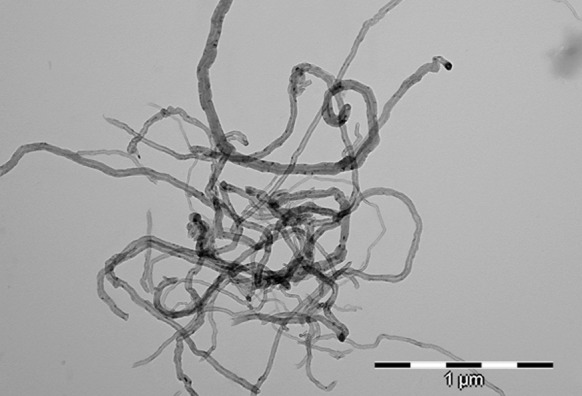
TEM photomicrograph of MWCNT

Raman spectroscopy is a sensitive technique for characterisation of carbon materials, including molecular allotropes of carbon. The Raman spectra of carbon nanoforms enable the differences in their structural features to be distinguished, and also those between the fullerenes and tubular structures referring to CNTs.

**Fig. 7:**
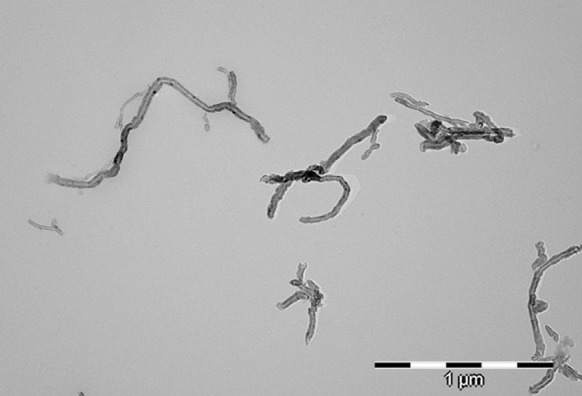
TEM photomicrograph of MWCNT after chemical functionalisation (MWCNT-F)

**Fig. 8:**
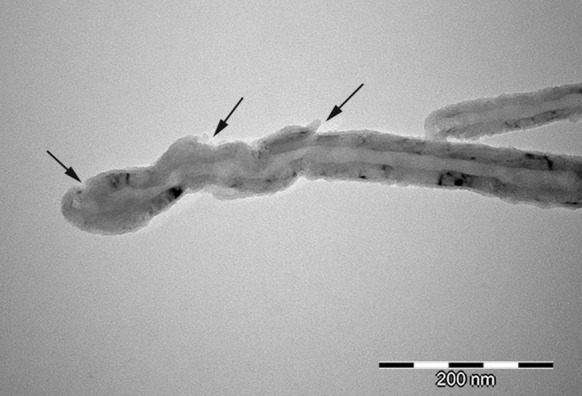
TEM photomicrograph of MWCNT after chemical functionalisation (MWCNT-F)

The Raman spectra of non-functionalised CNTs are shown in Fig. 5a. The spectrum of carbon structure contains two main bands: the G-band (1,606 cm^−1^—MWCNT), which is assigned to the E2g C–C (sp^2^-bonded) stretching mode of a well-ordered graphitic structure and the D-band (1,312 cm^−1^ for MWCNT), attributed to the A1g (sp^3^-bonded) stretching mode, resulting from the presence of a disordered structure or lattice defects in the graphite structure (substitutional heteroatoms, vacancies or chemically bonded heteroatoms). The intensity ratio of the D-line (ID) to the G-line (IG) in Raman spectra is a useful parameter to evaluate the structural ordering of carbon materials, including carbon nanoforms. A high ID/IG ratio means the presence of defects inside the carbon layers. This parameter equals 2.0 for MWCNTs. This high value for MWCNTs indicates that the degree of crystalline perfection for this type of nanotube is distinctly lower. The bands at 2,623 cm^−1^ (G′ band) for MWCNTs constitute an overtone of the D band. Due to such a highly defective MWCNT structure, it is more susceptible to the acids’ oxidative mixture. The length of MWCNT after chemical treatment (MWCNT-F) generally decreased (Fig. 7) in comparison to the pristine MWCNT (Fig. 6). Moreover, some surface changes such as destruction of the graphene layers were observed (Fig. 8, arrows). Such changes were not observed for as-prepared MWCNT.

### Size distribution of CNTs

**Fig. 9:**
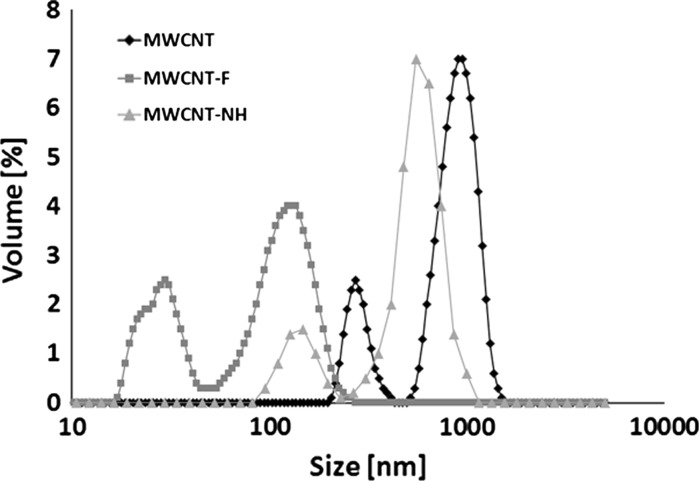
Size distribution of multi-walled carbon nanotubes before (MWCNT) and after oxidation (MWCNT-F) and functionalisation in ethylenediamine (MWCNT-NH)

In order to visualise differences between MWCNT before and after chemical oxidation in concentrated acids and functionalisation in ethylenediamine, the dynamic light scattering (DLS) method was used (Fig. 9).

Figure 9 compares the agglomerate size distributions of CNTs in water suspension preparations through sonication with ultrasound, for 15 min, using PALMER INSTRUMENTS (Model: CP 130 PB, 130 W power, 20 kHz). Bimodal distribution of MWCNT before oxidation was observed (mean values: 270 and 900 nm, respectively) (Fig. 9). The size distribution of MWCNT-F (after oxidation) was completely different in comparison with pristine MWCNT (Fig. 9). These results confirm the influence of the chemical treatment on the dispersion of carbon aggregates in water. The size distribution of MWCNT-F is shifted towards lower values. The mean sizes of MWCNT-F were 29 and 124 nm, respectively. The presence of carboxyl groups on the CNT surface change their wettability from hydrophobic to hydrophilic, which is demonstrated by facilitating the dispersion of nanotubes in an aqueous solution. Another very important observation after oxidising treatment is the reduction of the length of the CNTs. The size distribution of MWCNT-NH is different in comparison to oxidised MWCNT (MWCNT-F), though a more similar to as-prepared MWCNT. The mean sizes of MWCNT-NH are 148 and 553 nm, respectively. After functionalisation of MWCNT-F in ethylenediamnie the C=O bonds of the carboxyl groups are replaced by amine group resulting in the surface chemistry changes of nanotubes. Therefore, the nanotubes lose their affinity to the aqueous solution, resulting in poor dispersity and a stronger tendency to re-agglomeration.

### In vitro investigation of CNTs

For assessment of cytotoxicity and cell transport of different types of CNTs, the macrophage cell line (RAW 264.7) was used. Before cells were exposed to the CNTs, all types of samples (MWCNT, MWCNT-F and MWCNT-NH) were immersed in PBS and dispersed for 2 min using a tip sonicator (PALMER INSTRUMENTS, Model: CP 130 PB). From each of the solutions containing MWCNT, MWCNT-F and MWCNT-NH, equal portions of these materials were transferred to cells in culture medium. Two concentrations of CNTs, i.e. 38 and 80 μg/ml were used for experiments.

**Fig. 10:**
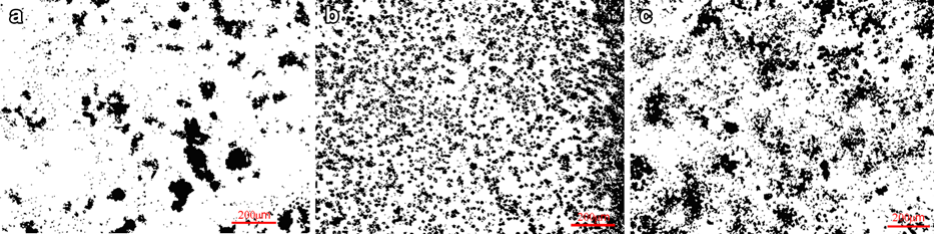
Dispersion of CNTs in culture medium with cells [MWCNTs (**a**), MWCNT-Fs (**b**), MWCNT-NHs (**c**))

Depending on the type of chemical treatment, the CNTs behaved in different ways in solution. The best dispersal was observed for MWCNT-F. The presence of carboxyl and hydroxyl groups on the CNT surface makes them more hydrophilic. Another factor influencing a better dispersion of MWCNT-F in buffer is their dimension. The MWCNT-Fs with a shorter length have a lower tendency to create big agglomerates and are easier to use to prepare a homogenous suspension. The stability of various solutions containing nanotubes has been confirmed by measuring their zeta potentials (ζ). This parameter for MWCNTs, MWCNT-Fs and MWCNT-NHs was −24.7, −60.9 and +15.3 mV, respectively. According to standard (ASTM 1985), if the absolute value of ζ is smaller than ±25 mV, the repulsive force is not strong enough to overcome the van der Waals attraction between the particles, and hence, the particles agglomerate (Castro et al. 2009). Therefore, the results obtained, especially for MWCNT-F, suggest that the nanotubes are well dispersed. The differential dispersion of CNTs in cell culture is illustrated in photomicrographs in (Fig. 10).

**Fig. 11:**
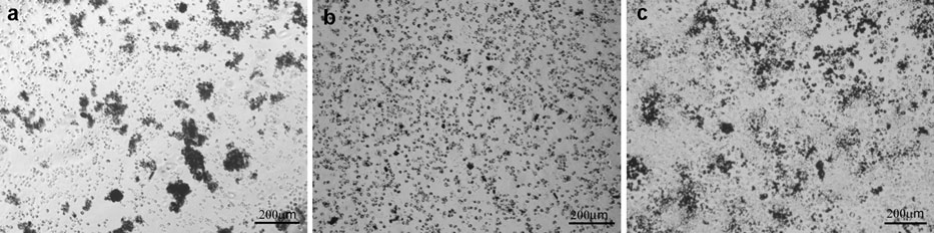
CNTs in contact with macrophages [MWCNTs (**a**), MWCNT-Fs (**b**), MWCNT-NHs (**c**)]

**Fig. 12:**
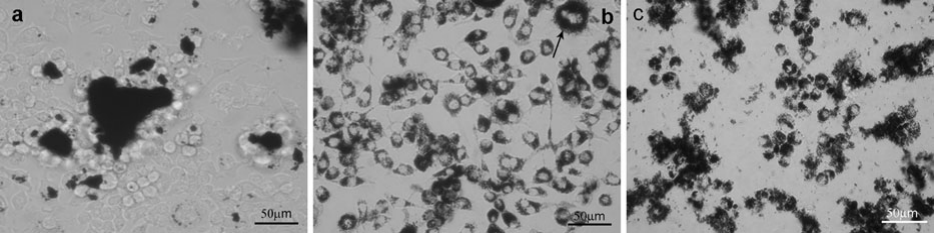
CNTs in contact with macrophages [MWCNTs surrounded by multinucleated cells (**a**), MWCNT-Fs inside macrophages (**b**), MWCNT-NHs in contact with macrophages (**c**)]

**Fig. 13:**
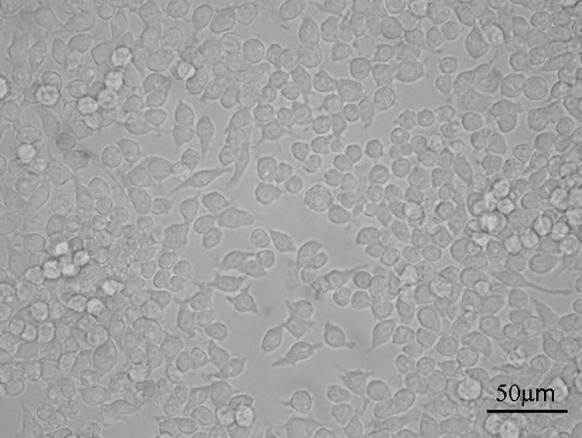
Control sample—macrophages without CNTs

As observed in Fig. 10b, MWCNT-F created the smallest aggregates with a regular shape because the majority are located in cells, which are more visible in Figs. 11b and 12b. The MWCNTs and MWCNT-NHs dispersed poorly in culture medium (Fig. 10a, c), which is especially noticeable for CNTs without chemical modification. Large agglomerates of nanotubes with strange shapes were observed for MWCNT (Fig. 11a). The existence of such agglomerates caused a different mechanism of interaction between the cells and CNT material. In this case, the likely phagocytosis process of nanotubes or direct transport through macrophage cells membrane was hindered. Moreover, for MWCNTs agglomerates the frustrated phagocytosis process was observed to be similar to that taking place in both in vivo and in vitro conditions (Fraczek et al. 2008; Castro et al. 2009; Shvedova et al. 2012; Cheng et al. 2009). When macrophage phagocytosis was hindered, giant cells surrounding the materials were observed (Fig. 12a). The morphology of macrophages without CNTs is presented in Fig. 13.

**Fig. 14:**
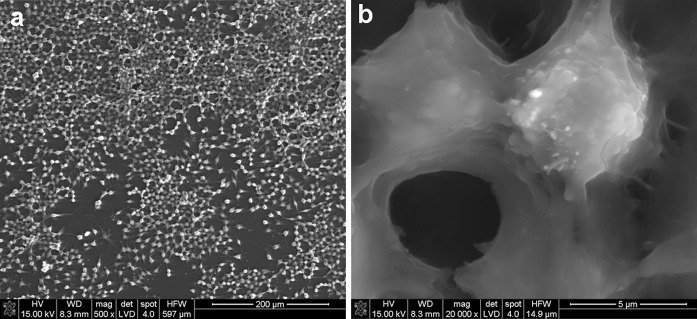
SEM photomicrographs of cells in control sample. Magnification ×500 (**a**), ×20,000 (**b**)

**Fig. 15:**
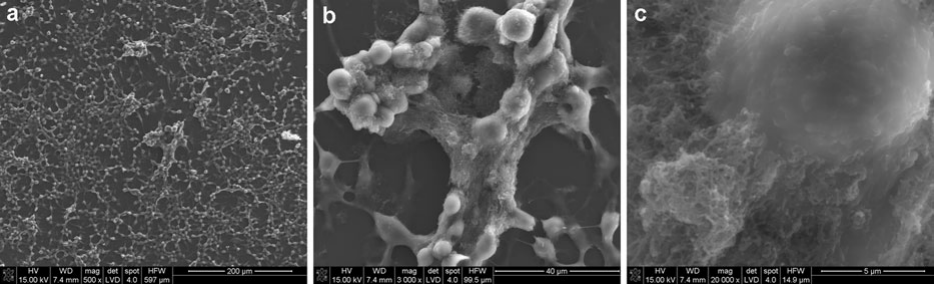
SEM photomicrographs of cells in contact with MWCNTs. Magnification ×500 (**a**), ×3,000 (**b**), ×20,000 (**c**)

**Fig. 16:**
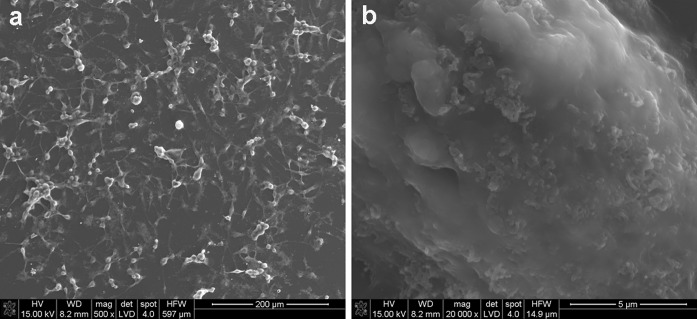
SEM photomicrographs of cells in contact with MWCNT-Fs. Magnification ×500 (**a**), ×20,000 (**b**)

**Fig. 17:**
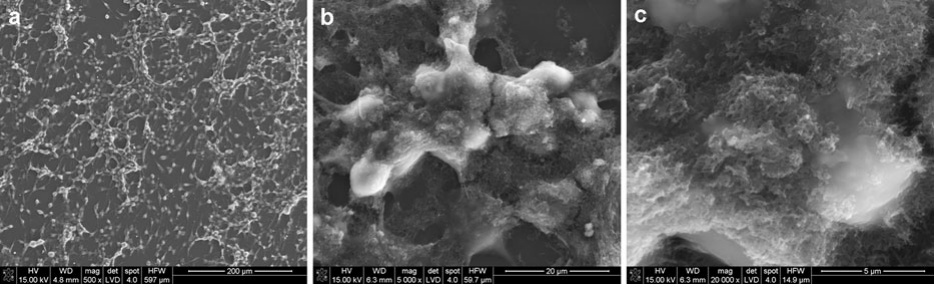
SEM photomicrographs of cells in contact with MWCNT-NHs. Magnification ×500 (**a**), ×3,000 (**b**), ×20,000 (**c**)

The interaction between MWCNTs and macrophages was also analysed using SEM (Figs. 14, 15, 16, 17). Depending on the types of CNT, a major difference was observed in the morphology of cells. The morphology of macrophages, especially in contact with MWCNTs (Fig. 15), was comparable to cells in the control sample (Fig. 14) (macrophages without CNTs). For this sample, the cells have a round shape in contrast to macrophages in contact with MWCNT-F, where cells have an elongated and fusiform shape (Fig. 16). For samples containing MWCNT-NHs macrophages, the majority display a round shape although fusiform shape cells were observed as well (Fig. 17). In the case of MWCNTs and MWCNT-NHs, the numbers of cells were higher than in cultures containing MWCNT-Fs. One of the reasons might be the presence of CNT agglomerates, especially in the samples containing MWCNTs. In this case, the majority of nanotubes accumulate to form agglomerates and thus leave the areas “free” of these materials so that the cells can proliferate as in the control sample. A better dispersion of MWCNT-Fs and difference in their shape in comparison to as-prepared MWCNTs make this material easier to transfer into the cells. It is also probable that the processes associated with transport of CNTs into the cells, e.g. phagocytosis, are more active than those connected with cell division. Another reason for the lowering of cell proliferation and distortion in cell shape for cells in contact with MWCNT-F, in comparison with MWCNTs and the control sample, may be a negative impact of nanomaterials on the normal physiological processes of these cells. Cells with apparently damaged membranes after contact with the purified nanotubes (MWCNT-F) were found under microscopy (Fig. 12b, arrow). Such a case was noted for cells filled with a large amount of CNTs. The presence of MWCNT-Fs in the cytoplasm of cells is shown by the SEM photomicrograph (Fig. 16b). The photomicrograph clearly shows that MWCNT-Fs are located across the cells membrane, which confirms the presence of nanotubes inside the cells. A similar behaviour of nanotubes has also been reported in the literature (Hu et al. 2010; Hirano et al. 2008).

For samples containing CNTs in the form of aggregates, the majority of cells were covered with them or located on them (Figs. 15b, c, 17b, c).

**Fig. 18:**
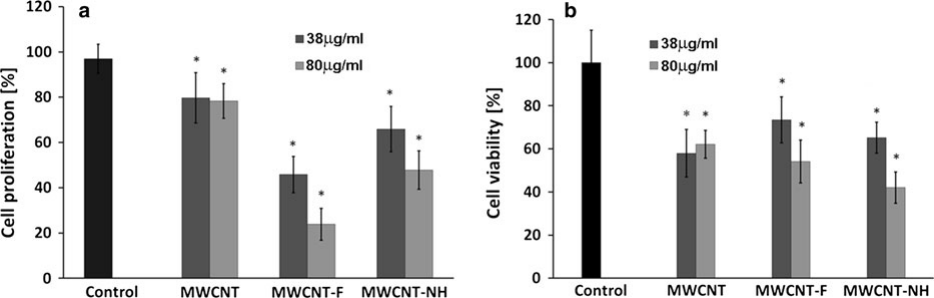
Cell proliferation (**a**) and viability (**b**) in contact with CNTs after 3 days of incubation. The dates are expressed as mean ± SD from 8 to 11 measurements, Student’s *t* test. *Statistically significant difference compared to controls (cell without CNTs = tissue culture polystyrene) (*p* < 0.05)

Analysis of cell proliferation in contact with the different kinds of CNTs after 3 days of culture revealed significant differences (Fig. 18a). The cell number for cells in contact with CNTs was lower in comparison with control samples (cells without nanomaterials). The lowest cell proliferation was observed for MWCNT-F, whereas the highest one was observed for MWCNTs. MWCNTs functionalised in ethylenediamine (MWCNT-NH) were found to have a higher number of cells than MWCNT-F, but lower in comparison to as-prepared CNTs (MWCNTs).

Analysing the impact of the nanotube concentration on cell proliferation revealed the significant difference for samples of both oxidised (MWCNT-F) and functionalised (MWCNT-NH). For both the samples, cell proliferation decreased with increasing concentration of CNTs. The results may suggest that MWCNT-Fs have a predominantly adverse impact on cell response. The physicochemical traits of these samples such as dimension, surface chemistry, dispersal and behaviour in cell culture observed both under optical and SEM may help to explain their effects on cell proliferation. MWCNT-F interact with the cells much more easily than the other samples by two mechanisms, namely, endocytosis/phagocytosis and an energy-independent passive process. Although the MWCNT-F sample produces the greatest reduction in cell proliferation of the macrophages compared to the control condition (Fig. 18a), according to the ToxiLight and ViaLight tests, it does not exhibit the highest toxicity compared to control (Fig. 18b). The results obtained from this test show that the cell viability for this sample is not lower than that obtained for MWCNT and MWCNT-NH. For lower concentrations of MWCNT-F (38 μg/ml), the cell viability is higher in comparison to MWCNTs and MWCNT-NH. This indicates that the mechanism of cell growth in contact with MWCNT-F differs as compared to the other samples. The higher number of cells in direct contact with this kind of nanotube than that observed for MWCNTs and MWCNT-NHs is due to a better dispersion of these materials in culture medium. A high concentration of MWCNT-F in macrophages may decrease the cell proliferation process by interfering with the mitotic apparatus without negative consequences on cell viability.

Surprisingly, although the as-prepared MWCNTs produced the least reduction in cell proliferation with reference to control, the viability of cells exposed to this sample was substantially reduced with respect to control (Fig. 18b). A possible explanation is the presence of MWCNT’s agglomerates, which are surrounded by numerous cells attempting to phagocytose them (Fig. 12a). When phagocytes meet particles that are too large to be phagocytosed, a phenomenon called frustrated phagocytosis may occur, in which proteolytic enzymes and toxic substances are released out from the cell (Brown et al. 2007; Anderson et al. 2008; Sanchez et al. 2011). These substances can damage the surrounding cells. Another reason related to a decrease in the cell viability for MWCNT is their length. High aspect ratio nanoparticles can induce frustrated phagocytosis and formation of multi-nucleated giant cells similar to the response of macrophages to asbestos fibers or CNTs following pharyngeal aspiration or interaperitoneal or pleural injection (Shvedova et al. 2012; Cheng et al. 2009; Sanchez et al. 2011; Porter et al. 2010; Donaldson et al. 2010; Mercer et al. 2010).

According to our previous works and the results presented by other authors the influence of metal residuals (catalysts used for CNT manufacture) on in vitro cellular response is significantly lower than effect of the length of the nanotubes and their agglomerated form (Poland et al. 2008; Fraczek et al. 2008; Sanchez et al. 2011; Zhao and Liu 2012). Impurities, including metallic catalyst particles within the CNTs, have been suggested to serve as a catalyst for oxidative stress. Negative influence of metallic catalysts on cells response is mainly observed for iron (Kagan et al. 2006; Guo et al. 2007; Hiraishi et al. 1991). Nickel was found to have harmful impact on the cells response; established human carcinogen that induces gene silencing and hypoxia signalling through mechanisms involving intracellular nickel cation (Liu et al. 2007). However, the influence of metal catalyst on cells response depends significantly on its concentration. In our samples, nickel content is 1.2 wt% for MWCNT and 0.1 wt% for MWCNT-F, i.e. its concentration is relatively low. Moreover, a part of the metal residue is entirely covered with CNT carbon graphene layers, which may also prevent its negative effect on the cellular response. Thus, metal residue in our samples has a negligible effect on the early cell response. A more significant effect of metal residue may be expected after a longer time of contact of CNT with cells culture. Thus, major factors affecting cells proliferation and their viability after 3-day culture are the presence of agglomerated forms of nanotubes and their length. It cannot be excluded, however, that negative effect of the metallic residues will reveal after a longer contact time with cells.

The cell viability level in contact with CNTs functionalised in ethylenediamine (MWCNT-NH) is between the viability of MWCNT and MWCNT-F. This result indicates that the presence of amine groups has no significant influence on cell behaviour at a low concentration of CNTs (38 μg/ml). In this case, the most important factor in cellular response is the dispersion of CNTs.

However, the concentration of CNTs is not without significance on cell viability. The decrease in cell viability with increase in CNT concentration (80 μg/ml) is especially visible for nanotubes after oxidation (MWCNT-F) and functionalisation (MWCNT-NH) processes.

## Conclusion

Three types of MWCNTs were investigated in this study. As-prepared CNTs (MWCNTs) were purified using concentrated acids (MWCNT-F) and then functionalised using ethylenediamine (MWCNT-NH). The results revealed that the same materials (MWCNTs) treated chemically may have a different influence on macrophage response. The importance of knowledge was proved concerning the physicochemical properties of the material to interpret the results of biological research. The treatment of CNTs in acidic medium affects their purity and introduces carboxyl groups on their surface. This process can also lead to changes in the dimensions of nanotubes. These factors in turn have a significant impact on their behaviour in culture media and consequently on the cellular response. A shorter and more hydrophilic MWCNT-F is more easily transferred inside the cells, probably through the phagocytosis process and free passage by cell membrane as compared to as-prepared MWCNTs and nanotubes in the form of agglomerates. The presence of CNTs inside the cells changes their morphology and slows down the processes of cell proliferation. At higher concentrations (up to 80 μg/ml) of this type of nanotubes, an adverse effect on cell viability was observed. Similar behaviour was observed for the nanotubes containing amino groups (MWCNT-NH). The presence of CNTs agglomerates distinctly decreases cell viability as a result of frustrated phagocytosis.
